# Organization and evolution of transposable elements along the bread wheat chromosome 3B

**DOI:** 10.1186/s13059-014-0546-4

**Published:** 2014-12-03

**Authors:** Josquin Daron, Natasha Glover, Lise Pingault, Sébastien Theil, Véronique Jamilloux, Etienne Paux, Valérie Barbe, Sophie Mangenot, Adriana Alberti, Patrick Wincker, Hadi Quesneville, Catherine Feuillet, Frédéric Choulet

**Affiliations:** INRA UMR1095 Genetics, Diversity and Ecophysiology of Cereals, 5 chemin de Beaulieu, 63039 Clermont-Ferrand, France; University Blaise Pascal UMR1095 Genetics, Diversity and Ecophysiology of Cereals, 5 chemin de Beaulieu, 63039 Clermont-Ferrand, France; INRA-URGI, Centre de Versailles, Route de Saint Cyr, 78026 Versailles, France; CEA/DSV/IG/Genoscope, 2 rue Gaston Cremieux, 91000 Evry, France; CNRS UMR 8030, 2 rue Gaston Crémieux, 91000 Evry, France; Université d’Evry, P5706 Evry, France

## Abstract

**Background:**

The 17 Gb bread wheat genome has massively expanded through the proliferation of transposable elements (TEs) and two recent rounds of polyploidization. The assembly of a 774 Mb reference sequence of wheat chromosome 3B provided us with the opportunity to explore the impact of TEs on the complex wheat genome structure and evolution at a resolution and scale not reached so far.

**Results:**

We develop an automated workflow, CLARI-TE, for TE modeling in complex genomes. We delineate precisely 56,488 intact and 196,391 fragmented TEs along the 3B pseudomolecule, accounting for 85% of the sequence, and reconstruct 30,199 nested insertions. TEs have been mostly silent for the last one million years, and the 3B chromosome has been shaped by a succession of bursts that occurred between 1 to 3 million years ago. Accelerated TE elimination in the high-recombination distal regions is a driving force towards chromosome partitioning. CACTAs overrepresented in the high-recombination distal regions are significantly associated with recently duplicated genes. In addition, we identify 140 CACTA-mediated gene capture events with 17 genes potentially created by exon shuffling and show that 19 captured genes are transcribed and under selection pressure, suggesting the important role of CACTAs in the recent wheat adaptation.

**Conclusion:**

Accurate TE modeling uncovers the dynamics of TEs in a highly complex and polyploid genome. It provides novel insights into chromosome partitioning and highlights the role of CACTA transposons in the high level of gene duplication in wheat.

**Electronic supplementary material:**

The online version of this article (doi:10.1186/s13059-014-0546-4) contains supplementary material, which is available to authorized users.

## Background

First discovered in maize [1], transposable elements (TEs) are ubiquitous components of almost every eukaryotic genome investigated so far and their impact on genome structure and evolution is now well established (reviewed in [2]). Two classes of TEs have been defined: class I, or retrotransposons that use the element-encoded mRNA as a transposition intermediate; and class II, or DNA transposons that excise from their insertion site and transpose through a DNA intermediate. Among sequenced plant genomes, TE abundance ranges from 20% in *Arabidopsis thaliana* [3] to 85% in maize (*Zea mays*) [4]. Genome expansion is mainly mediated by the activity of class I elements, while the content of DNA transposons is much more constant [5-7]. Furthermore, TEs are non-randomly distributed along the genome due to insertions [8,9] and deletions [7,10] that trigger genome expansion/contraction. Hence, in rice (*Oryza sativa*) [11], sorghum (*Sorghum bicolor*) [12] and maize, long terminal repeat retrotransposons (LTR-RTs) accumulate preferentially in heterochromatin, such as centromeric regions, and are less abundant in the recombinogenic distal parts of chromosomes. A molecular mechanism responsible for the targeted integration of TEs in heterochromatin has begun to be understood [13]. A key component of the TE integration complex is a chromodomain present at the carboxy-terminal part of the element-encoded integrase in some *gypsy* elements [14] that drives preferential insertion by targeting specific chromatin modifications [15]. Deletion of TEs can be identified by the identification of solo LTRs and truncated elements [6]. Presumably, solo LTRs are formed by unequal intra-chromosomal homologous recombination between two LTRs of an intact element [16]. In contrast, truncated elements are thought to be the outcome of illegitimate (nonhomologous) recombination. In rice, 190 Mb of LTR-RT DNA have been removed recently by these two processes, leaving a current genome of approximately 400 Mb that contains <100 Mb of detectable LTR-RTs [17]. In sorghum and rice, DNA transposons are localized essentially in the telomeric regions of the chromosome [11,12]. However, no evidence for a direct relationship between genetic recombination rate and DNA transposon abundance has been provided so far in plant genomes [18], whereas it was observed in *Caenorhabditis elegans* [19]. A key aspect of DNA transposons is their interaction with host genes [20]. For instance, they were shown to be involved in the creation of new genes through ‘exon shuffling’. In maize, 60% of the 20,000 Helitrons contain captured gene fragments [21,22]. Similarly, in rice, 2,809 Pack-Mutator-like elements (Pack-MULEs) containing host gene fragments were identified [23].

Identification of transposable elements in large and complex genomes is a daunting task, even in high quality assembled genomes. As an example, since 2001, in the rice genome, the number of miniature inverted-repeat TEs (MITEs) discovered has steadily increased over time from 6,641 to 179,415 [24-28]. A high-quality TE prediction and annotation is essential to prevent mis-annotation of functional genes and to understand the biology of genomes [29]. Different strategies have been developed, including similarity search against databanks of known TE sequences, *de novo* repeat detection, k-mer-based counting, and structural motif detection (reviewed in [30]). Despite the development of many dedicated bioinformatic tools, precise TE modeling in complex (>1 Gb) genomes, such as in wheat or maize, is a *tour de force*. During the maize genome sequencing project (approximately 2 Gb), TEs were predicted with a variety of approaches: LTR-RTs and Helitrons were identified by structural criteria, while the rest of the TEs were annotated by similarity with a library built by *de novo* detection [4]. One of the main limitations to identifying TEs, and especially large TEs (>5 kb), was the fragmentation of the assembly with many gaps and a short median contig size (approximately 7 kb).

The wheat genome is large and highly complex (17 Gb, allohexaploid 2n = 6x = 42 with 3 closely related subgenomes, AABBDD). Previous small-scale analyses revealed that the wheat genome is composed of about 80% TE-derived sequences, mainly nested into each other and with a few families representing 50% of the TE fraction [31]. Early, manually curated TE modeling was performed on selected bacterial artificial chromosome (BAC) sequences during map-based cloning projects or using plasmid or BAC end sequences [32-36]. At a larger scale, analyses of 18 Mb of long BAC contig sequences spread along the 3B chromosome [31] led to precise delineation of 3,222 TEs. Together with the public *Triticeae* REPeat sequence database (TREP) [37], this provided a representative high quality reference library of wheat TEs. Beyond the identification of TEs, the reconstruction of the nested insertion pattern is a computational challenge that requires fine tuning of dedicated algorithms and knowledge of TE structure and evolution.

Recently, a number of genome scale sequences were produced from different wheat genomes. Whole genome shotgun sequencing using short read sequencing technologies has been performed on bread wheat and diploid species related to the homoeologous A and D genomes [38-40]. While these sequences have been useful to characterize the gene space, the assemblies were highly fragmented and therefore had only limited value for studying TEs. Moreover, they do not provide sufficient sequence contiguity to assemble pseudomolecules, precluding the analysis of any TE feature distribution along a chromosome. To obtain a reference genome sequence of bread wheat, the International Wheat Genome Sequencing Consortium (IWGSC) [41] has established an approach based on flow sorting of individual chromosomes and the construction and sequencing of chromosome-specific physical maps. Recently, we produced a pseudomolecule for the largest wheat chromosome (3B), which represents 774 Mb, carrying 7,264 genes and 85% of TE-derived sequences [42]. Gene annotation and comparative analyses indicated that chromosome 3B, and the wheat genome in general [43], carries a higher number of genes than related grass species. Moreover, the results showed that about 35% of the 3B genes share similarity with genes located on non-orthologous chromosomes in other grasses [42]. These ‘nonsyntenic’ genes likely originate from interchromosomal duplications triggered by diverse mechanisms such as double strand break repair or TE mobilization.

In this study, a strategy dedicated to TE-modeling in a complex genome was developed to decipher the complex organization of TEs along a wheat chromosome. Analyses of the distribution of the abundance, diversity and dynamics of TEs revealed a striking partitioning of the chromosome, as observed for other features on chromosome 3B [42]. In addition, we observed a massive amplification of CACTA DNA transposons compared with other related grass species with a significant association between some CACTA families and recently duplicated genes, suggesting a role for CACTA transposons in gene duplications, gene capture, and genome plasticity in wheat.

## Results

### An improved procedure for predicting transposable element models and their nested pattern in a complex genome

Predicting TE features in complex genomes where repeated elements represent more than 80% of the sequence remains a computational challenge, and obtaining a high quality annotation still requires manual curation. Typically, TE prediction is performed by similarity search with a set of known TE sequences. A major prerequisite to achieve a high quality annotation is the availability of a curated TE reference library. This is essential to identify most transposons via similarity search-based approaches and to restrict the *de novo* detection of repeats to the unassigned portion of the genome sequence. In wheat, two curated libraries dedicated to *Triticeae* (wheat, barley and rye) TEs are available: TREP, which contains 1,717 TEs representing 323 families, and an additional set of 3,212 TEs manually annotated in a previous pilot study of chromosome 3B [31]. For most of them, the borders of the mobile element have been defined precisely and their completeness - that is, ‘complete element’ versus ‘fragmented element’ - is also available. However, there are incongruencies in the classification of the TEs; most of the TEs were assigned a family name based on their best BLAST hit, which can lead to an overestimation of the family numbers [44].

To ensure proper TE modeling of the 774 Mb of sequence from chromosome 3B, we first built a classified TE library (ClariTeRep; see Materials and methods) using the 3,050 complete TE sequences from these two libraries. In total, 525 families, comprising 1 to 266 copies each, were clustered based on their sequence similarity. Among the families described in TREP, 40% (277/700) have a one-to-one relationship with the ClariTeRep classification while the others were mostly aggregated into a single family and, sometimes, split into different families. More than 80% of the TREP families comprise one to two TE copies only versus 57% for ClariTeRep, confirming that the similarity-based classification increases the number of families compared with the clustering-based approach [44].

In the second step, we automated two things: (i) correction of the over-fragmentation of TE predictions, that is, the fact that a TE is not detected as a single feature but rather split into several neighboring ones; and (ii) the reconstruction of nested TE insertions. To this aim, we developed a program called CLARI-TE, which allows merging neighboring predictions that belong to the same family (see Materials and methods). Then, nested clusters were automatically reconstructed by joining remote predictions belonging to a single element. We estimated the accuracy of the automated annotation by analyzing a manually annotated sequence of approximately 1 Mb (scaffold v443_0137) containing 196 TEs and 47 nested insertions that was manually annotated for this purpose. We compared the annotation produced by RepeatMasker (using ClariTeRep) with that from CLARI-TE and another annotation pipeline, TEannot (part of the REPET package [45]) (Table 1). At the nucleotide level, RepeatMasker correctly assigned approximately 90% of nucleotides that belong to TEs, showing that our TE library is comprehensive enough to detect the vast majority of TEs in a wheat genomic sequence. However, the 196 TEs were predicted as 590 separated features with RepeatMasker, illustrating the over-fragmentation problem. The improvement was significant when using both TEannot and CLARI-TE, which detected 345 and 286 features, respectively (Table 1). CLARI-TE was more accurate than TEannot in predicting the correct borders of the TEs (sensitivity of 66% versus 45%, respectively) and in limiting the number of false positives (specificity of 52% versus 27%, respectively). In addition, CLARI-TE was able to perform the reconstruction of nested clusters much more accurately than TEannot (Table 1). The accuracy of nested insertion mining was in fact highly dependent on the type of TEs: sensitivity and specificity were much higher for *gypsy* and *copia* elements (53% and 68%, respectively) than for CACTAs (13% and 40%, respectively). This suggests that CACTAs exhibit a higher level of sequence variability and that CACTA-typical short tandem repeats limit our ability to identify them through fully automated procedures.

**Table 1:** **Comparison of the accuracy of transposable element modeling on a 1 Mb scaffold of wheat chromosome 3B**

		**Reference**	**RM**	**TEannot**	**CLARI-TE**
Number of predictions		196	590	345	286
Coverage		91%	90%	90%	91%
Nucleotide	Sn	-	93%	95%	96%
	Sp	-	96%	97%	95%
Feature	Sn	-	54%	45%	66%
	Sp	-	26%	27%	52%
Nested feature	Sn	-	NA	17%	41%
	Sp	-	NA	14%	58%

The reference annotation was curated manually. Similarity search was performed using RepeatMasker (RM) and automated TE modeling based on the RM results was performed using either TEannot or CLARI-TE. The sensitivity (Sn) and specificity (Sp) were calculated at three different levels: nucleotide, feature and nested feature (see Materials and methods). NA, not applicable.

### Transposable element content and distribution along chromosome 3B

We used CLARI-TE to predict TE models along the 833 Mb of chromosome 3B (a sequence corresponding to the 3B pseudomolecule (774 Mb) and unanchored scaffolds (59 Mb)) in order to study the organization and dynamics of TEs along a wheat chromosome. In total, 523,233 RepeatMasker-detected features were combined with CLARI-TE to obtain a final set of 56,488 complete TEs (that is, with two borders corresponding to the borders of a reference element) and 196,391 truncated or partially assembled elements. Given the estimates of the sensitivity and specificity of our approach to predict complete TEs, we can extrapolate to approximately 73,000 the number of intact TEs carried by the 3B chromosome. Thus, using CLARI-TE we are able to reduce the fragmentation of the annotation by two-fold and detect the largest set of full-length TEs and nested clusters ever observed on a plant chromosome so far. The number of large TEs (>5 kb) was doubled (from 16,988 to 30,894) when using CLARI-TE. Moreover, 30,199 nested insertions were reconstructed, comprising up to 8 layers and 320 clusters larger than 100 kb (and up to 301 kb). Overall, TEs represent 85% of the 833 Mb of chromosome 3B scaffolds. In addition, the de novo identification revealed 3% of previously uncharacterized TEs [42]. We applied the same approach to annotate the draft genome sequences of the A- and D-related diploid progenitors: *Triticum urartu* (approximately 3 Gb) [39] and *Aegilops tauschii* (approximately 2.6 Gb) [40]. TEs account for 77% and 74% of the A and D diploid genome assemblies, respectively (Table S1 in Additional file 1). We also found the proportion of complete TEs to be 12% and 11%, respectively, that is, two times lower than on chromosome 3B (22%).

**Figure 1:**
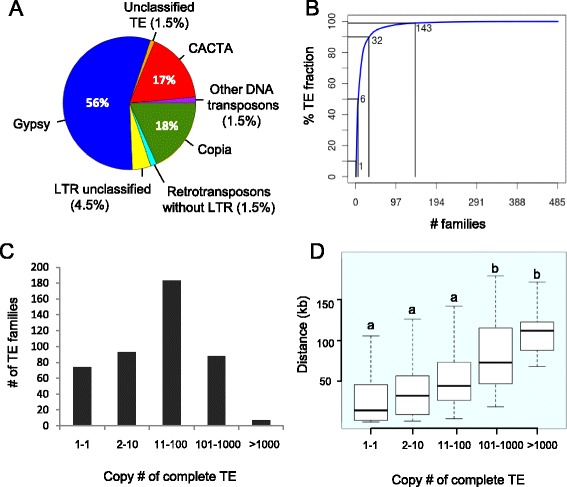
**Transposable element content and copy number of the wheat chromosome 3B sequence. (A)** Pie graph of the relative composition of the main TE superfamilies. **(B)** Cumulative sum of the number of TEs families among the TE fraction. The N50 is 6, meaning that six TE families represent 50% of the TE fraction (in number of nucleotides). **(C)** Distribution of the number of copies per family (considering complete copies only). **(D)** Box plot of the distance (in kilobases) of TEs to the closest gene. The five categories represent TE families with different numbers of copies on the 3B chromosome.

The annotation revealed that class I and II TEs represent 67% and 18% of the 3B sequence, respectively, with a vast majority of the chromosome corresponding to LTR-RTs (529 Mb, 66% of the chromosome; Table S1 in Additional file 1). Three superfamilies (*gypsy*, 47%; CACTA, 16%; *copia*, 16%) account for more than 79% of the total TE fraction (Figure 1A). This proportion of CACTAs is much higher than in the other sequenced grasses: 3.2% in maize [4], 4.7% in sorghum [12], 3.4% in rice [11], and 2.2% in *Brachypodium distachyon* [46]. We also found a higher proportion of CACTAs in the draft genome sequences of *T. urartu* and *Ae. tauschii* (12.3% and 15.6%, respectively; Table S1 in Additional file 1), suggesting that most of the CACTA amplification occurred before the divergence of the A, B and D genomes. Other DNA transposons are less abundant in terms of proportion but some correspond to small size elements found in very high copy numbers. For example, 17,479 MITEs (clustered into 95 families) of, on average, 142 bp, mostly from the *Mariner* superfamily, were detected along the 3B chromosome sequence.

In total, 485 families were detected along the 3B chromosome with only 6 families representing 50% of the TE fraction, and 143 representing 99% of the TE fraction (Figure 1B). The most abundant element was the LTR-RT RLG_famc1 (Fatima) family with 7,036 complete and 8,003 fragmented copies that account on their own for 12% of the TE fraction. Fifteen percent (74) of the families are represented by a single copy element on the entire chromosome 3B sequence while 2% (7) have amplified into more than 1,000 copies. Therefore, most of the TE families are not single copy member families but have rather amplified at a medium copy number (between 11 and 100) within this chromosome (Figure 1C). This pattern is different from what has been described for related grasses where single copy member families represent between 66% and 81% of the families in rice, *Brachypodium*, sorghum, and maize [44]. We evaluated the impact of the classification methods on these findings by comparing the average percentage of identity between members of the LTR-RT families for the different grass genomes (Figure S1 in Additional file 1). Our clustering approach resulted in an average of 77% identity over the full length of TEs while this percentage was higher (ranging between 86% and 89%) in the classification of El Baidouri *et al*. [44] for *Brachypodium*, rice, sorghum, and maize. Applying a cutoff at 77% identity showed that the proportion of single copy member families decreases drastically in the related grasses (16%, 52%, 25%, and 17% in rice, *Brachypodium*, sorghum and maize, respectively), leading to a distribution similar to that observed in wheat. When calculating the distance between each TE and its neighboring genes we observed that low-copy number families are significantly closer to genes than highly repeated families (one-way ANOVA, *P*-values <4.5e^-11^; Figure 1D). Indeed, families with less than 100 copies were found to be significantly closer to genes than families having more than 100 copies (Bonnferroni/Dunn test, *P*-values <4e^-4^).

**Figure 2:**
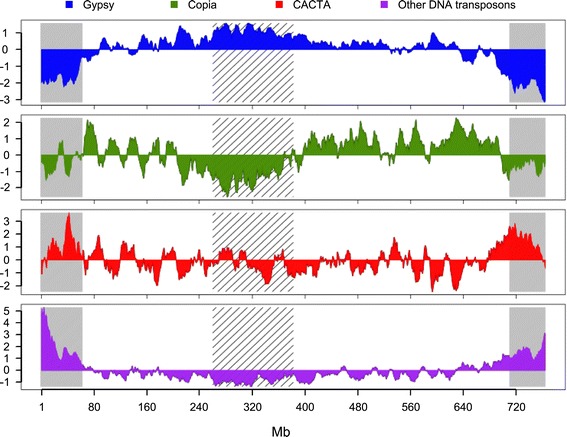
**Distribution of variations of transposable element density along wheat chromosome 3B.** Distributions are represented for four superfamilies: *gypsy* (blue), *copia* (green), CACTA (red), and other DNA transposons (purple). The distributions were calculated in a sliding window of 10 Mb with a step of 1 Mb and the graphs represent the standard score, that is, variations around the mean in number of standard deviations. Recombinogenic distal and centromeric/pericentromeric regions [42] are labeled by grey and hatched areas, respectively.

Overall, the TE distribution along the chromosome is strongly correlated with that of the *gypsy* elements (R = 0.97) and is negatively correlated with the recombination rate (R = -0.82, *P*-values <10e^-10^) (Table S2 in Additional file 1). The CACTA superfamily exhibits the exact opposite pattern, with a significant increase of 23% in the distal regions (18 to 19%) compared with the proximal region (15%; Figure 2). Similar to CACTAs, but at a lower level, class II transposons - Harbingers, hATs, Mariners, Mutators, and Helitrons - are twice as abundant in the distal regions compared with the proximal region and their distribution is strongly correlated with both the gene density (R = 0.63) and recombination rate (R = 0.71).

The distribution of TE diversity - that is, the number of different families per 10 Mb - along the chromosome was also investigated. It revealed a higher diversity in the distal regions (210 different TE families per 10 Mb on average, maximum of 244 families within 10 Mb) than in the central part of the chromosome (134 different families per 10 Mb; Figure S3 in Additional file 1). The diversity of DNA transposons increases by 2.6-fold in the distal region compared with the centromere whereas the diversity in LTR-RTs is homogeneous along the chromosome (106 ± 7 families per 10 Mb; Figure S3 in Additional file 1). It is worth noting that the patterns of distribution of TE diversity and TE density are opposite. Thus, the increase in the amount of TEs in the centromeric regions is due to the accumulation of several copies from the same families.

Segmentation analysis (see Materials and methods) of TE density variations along the chromosome showed the presence of five distinct regions. The two chromosome ends, representing 18% of the chromosome (63 Mb and 73 Mb on the short and long arms, respectively), exhibit the lowest TE content (71%). The 122 Mb (16% of the chromosome region) encompassing the centromere [42] has the highest TE content (93%). Finally, the two core parts of the chromosome arms (66% of the chromosome; 200 Mb and 316 Mb for the short and long arms, respectively) exhibit an average TE content of 88%. The borders of the two distal TE-poor regions correspond almost exactly to the regions (R1 and R3) defined by the recombination pattern in [42].

### Uneven distribution and impact of transposable elements on the evolution of the chromosome structure

**Figure 3:**
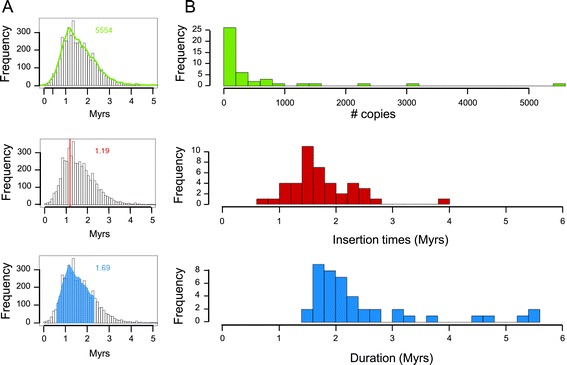
**Insertion date and period of amplification of LTR-RTs on wheat chromosome 3B. (A)** Distribution of the insertion dates for 5,554 complete copies of the RLG_famc1 (Fatima) family. The number of copies is highlighted in green (top), the peak of amplification is in red (middle), and the period of activity is in blue (bottom). **(B)** Distribution of the frequency of the copy number, insertion dates, and period of activity using 43 LTR-RT families with at least 20 copies.

**Figure 4:**
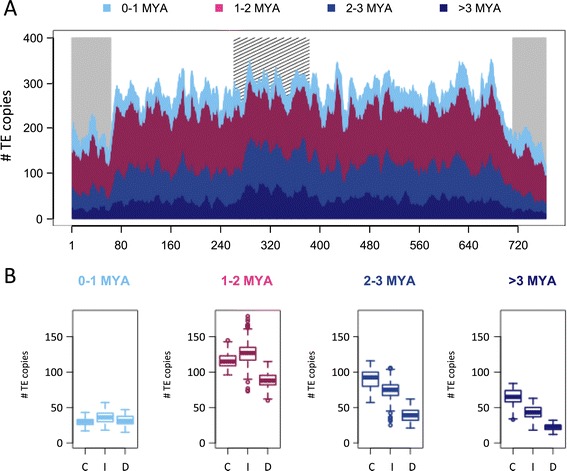
**Relationships between insertion dates and chromosomal locations of LTR-RTs.** In total, 21,165 LTR-RTs with an estimated insertion date have been grouped within four classes: 0 to 1 MYA (blue), 1 to 2 MYA (purple), 2 to 3 MYA (green), >3 MYA (red). **(A)** Distribution of the number of LTR-RTs for each of these four categories along chromosome 3B. Distribution was calculated in a sliding window of 10 Mb with a step of 1 Mb. Recombinogenic distal and centromeric/pericentromeric regions [42] are labeled by grey and hatched areas, respectively. **(B)** Box plot of the number of TEs per 10 Mb sliding window carried by the recombinogenic distal regions (D), the internal parts of the chromosome arms (I), and the centromeric/pericentromeric regions (C).

To investigate the evolutionary dynamics of LTR-RTs in chromosome 3B, we estimated the insertion dates of 21,165 intact copies. We analyzed the distribution of insertion dates of individual copies for each of the 43 families with at least 20 copies in order to retrieve a family-specific burst date and period of activity (Figure 3A). Our analysis revealed that 93% of the TE bursts occurred between 1 and 3 million years ago (MYA; Figure 3B), confirming at the whole chromosome scale that TE amplification has slowed down and/or that TE deletion has increased for the last 1 million years [31]. The active transposition periods (Figure 3B) lasted from 1 to 3 million years and correspond to a succession of bursts every 40,000 years on average. This indicates that the wheat genome has been shaped by successive waves of TE activation quickly followed by silencing. Indeed, 69% of the families were estimated to have been active over periods ranging from 1.5 to 2.5 million years. Only a few families were active for a longer period of time, suggesting they have escaped silencing. Not surprisingly, the intensity of the burst was negatively correlated with the amplification period (R = -0.4, *P*-values = 0.007), suggesting that the higher the level of activity the faster the silencing was established. Moreover, we performed in-depth analyses to gain deeper insight into the transpositional activity among widely expanded LTR-RT families. Dendrograms based on the multiple alignments of the full length LTR-RTs were computed for the RLG_famc1 and RLC_famc2 families (Figure S2 in Additional file 1). For RLG_famc1, this revealed the presence of 10 clusters indicating the presence of 10 master copies. Beside a global burst of transposition at 1.2 MYA, transposition bursts of each variant occurred between 0.9 and 3.0 MYA. A bimodal distribution was observed for RLC_famc2 with an old peak around 7 MYA and a recent one at 1.45 MYA. The dendrogram of the RLC_famc2 family revealed the presence of at least five master copies that have successively amplified, highlighting the fact that highly repeated families amplified via successive waves of transposition involving one or a few master copies that escape silencing. In order to investigate the type of evolutionary forces that have shaped the chromosome 3B structure, we studied the potential differences in chromosomal distributions of LTR-RTs depending on their insertion date. Four different categories were defined based on the TE insertion at <1 MYA, 1 to 2 MYA, 2 to 3 MYA, and >3 MYA (Figure 4). The analysis revealed that the gradual increase in the proportion of LTR-RTs from the telomeres to the centromere is in fact explained by an overrepresentation of ancient elements (>3 MYA). Indeed, no significant variation in the density of young (<1 Mya) LTR-RTs was observed along the chromosome, whereas enrichments of 1.3-, 2.3- and 2.8-fold were found in the proximal compared with the distal regions for TEs inserted 1 to 2 MYA, 2 to 3 MYA, and >3 MYA, respectively. These results suggest that new TE insertions occur at a similar rate along the chromosome and, consequently, that the gradient observed is due to a higher rate of elimination in the recombinogenic distal regions.

One of the prevalent mechanisms for LTR-RT elimination is the formation of solo-LTRs through ectopic homologous recombination between pairs of LTRs [16]. We detected 3,998 solo-LTRs with target site duplications on the chromosome 3B pseudomolecule, revealing a ratio of solo-LTRs to intact elements of 0.13:1, similar to what was observed previously in both wheat (0.14:1) [31] and maize (0.14:1) [44] but much lower than in rice (1.39:1) [44]. The second mechanism involved in TE turnover is illegitimate recombination that generates truncated TEs. A ratio of truncated to intact elements of 3.5:1 was found on the pseudomolecule, which is higher than the 0.5:1 ratio estimated previously for chromosome 3B [31]. This is mostly because of a higher number of gaps found in the assembly of the pseudomolecule versus a few BAC contigs whose sequence was completely finished that led to prediction of intact elements as truncated. A higher ratio of truncated versus intact elements (27:1) was estimated in maize due to an even higher number of gaps, especially in LTR-RTs, in a sequence that was mostly aimed at completing the gene space [47]. Nevertheless, given that the assembly quality is homogeneous along chromosome 3B, local variations in the truncated to intact ratio were investigated. This revealed a pattern very similar to the ratio of solo/intact elements (Figure S4 in Additional file 1) with a significant increase in telomeric regions compared with the proximal region (one-way ANOVA, *P*-value <0.001), again suggesting faster TE turnover in recombinogenic regions. Hence, the proportions of both solo-LTRs and truncated LTR-RTs are correlated with the recombination rate (R = 0.41 and R = 0.56, respectively; *P*-value <0.001).

Preferential insertion of large TEs could also play a role in the observed uneven distribution, since the chromodomain-containing integrase of LTR-RTs could specifically target heterochromatin [13]. Among the 68 *gypsy* families identified on the 3B pseudomolecule, 7 encode a chromodomain-containing integrase and their distribution is biased with an increase in the proximal region (Figure S5 in Additional file 1). The abundance of *gypsy* elements harboring a chromodomain increases by 80% in the proximal region while *gypsy* elements without chromodomains increase by 20%, suggesting that chromatin affinity during transposition may also have contributed to this pattern.

### Impact of CACTA transposons on the evolution of the gene content through gene duplication

**Figure 5:**
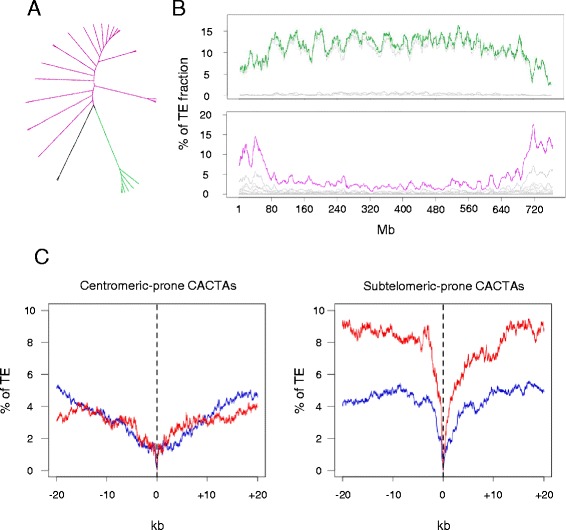
**Relationships between CACTA families and nonsyntenic genes. (A)** Tree based on the clustering of the distribution patterns of 30 CACTA families found along the 3B chromosome. The branches represented in purple and green correspond to subtelomeric-prone CACTA families and centromeric-prone CACTA families, respectively. **(B)** Distribution of the 6 centromeric-prone CACTA families (top) and 22 subtelomeric-prone CACTA families (bottom) showing opposite patterns along the 3B chromosome sequence. The gray curves represent the distribution of individual families and the top purple or green curves represent the cumulative sum of all families. **(C)** Abundance of centromeric-prone (left panel) and subtelomeric-prone (right panel) CACTAs in the vicinity (±20 kb) of syntenic (blue) and non-syntenic genes (red), respectively. 0 represents the position of the coding sequences (start and stop codons) and the average abundance of CACTAs was calculated for each nucleotide in a -20/+20 kb window encompassing the coding sequences.

Chromosome 3B carries at least 2,065 genes/pseudogenes that are nonsyntenic with the related model grass genomes of *B. distachyon*, rice, and sorghum [42]. These genes originated from recent interchromosomal duplications (after the divergence with *Brachypodium* 39 MYA [46]) and have preferentially accumulated in the distal regions of the chromosome. DNA transposon-mediated gene capture, which has already been suggested in wheat [31], was performed to estimate the relationship between the increases of both CACTAs and duplicated genes in the distal regions. In order to identify CACTA families significantly associated with the nonsyntenic genes, hierarchical clustering was applied to distributions of CACTA families (see Materials and methods; Figure 5A). We identified two groups containing 22 and 6 families (out of 30 families) with opposite patterns. The first group of 22 families was found to be overrepresented in the distal region (for example, the Caspar family), while the second one containing six families was more abundant in the proximal regions (for example, the *Jorge* family) (Figure 5B). Investigating potential site-specific insertion (within 50 bp around the elements) did not show any preferential insertion site for any of these groups. In addition, the two groups shared similar transposase domains. Distribution of subtelomeric-prone CACTAs was highly correlated with the distribution of nonsyntenic genes (R = 0.8). Then, we compared their frequency in the close vicinity (±20 kb) of nonsyntenic versus syntenic genes (Figure 5C). This revealed that subtelomeric-prone CACTAs are twice as frequent in the vicinity of nonsyntenic versus syntenic genes. By contrast, no difference was observed for the centromeric-prone CACTAs. For example, the DTC_famc5 (*Vincent*) family exhibited a six-fold increase in the promoter (5 kb upstream) region of nonsyntenic genes compared with syntenic genes.

We detected 140 CACTA-mediated capture events involving 145 genes and 11 CACTA families (see Materials and methods). The DTC_famc5 (*Vincent*) family accounted for 74% (104) of the cases. Captured genes were smaller (average coding sequence (CDS) size of 488 bp versus 1,090 bp) and with fewer exons (3.05 versus 4.11) than the average genes on chromosome 3B. Forty-six percent of them exhibited a structure likely to be functional while the others were classified as pseudogenes. Using expression data (RNAseq performed on five organs at three developmental stages each [48]), we showed that 26% (38) of the CACTA-captured genes were transcribed. The 145 gene copies captured represent 121 different families of genes. Interestingly, we found two cases where the same gene was captured twice by two different CACTAs. A putative 3′-5′ exonuclease encoding gene is repeated in three copies of the DTC_famc5 (*Vincent*) family (TRAES3BF090200030CFD_t1, TRAES3BF060400250CFD_t1, TRAES3BF060000130CFD_t1) and in seven copies of the DTC_famc6 family (*TAT*) (TRAES3BF032300010CFD_t1, TRAES3BF032400050CFD_t1, TRAES3BF067500010CFD_t1, TRAES3BF077700050CFD_t1, TRAES3BF082000030CFD_t1, TRAES3BF168400140CFD_t1, TRAES3BF182300010CFD_t1). The high level of conservation between the captured segments suggests that these two families have recently exchanged DNA.

**Figure 6:**
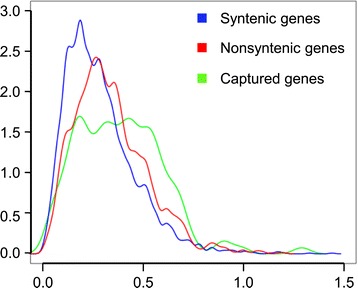
**Selection pressure estimated by the dN/dS ratio for CACTA-captured genes.** Distribution of the frequency of the dN/dS ratio for 2,964 syntenic genes (blue), 1,179 nonsyntenic genes (red) and 127 CACTA-captured genes on chromosome 3B (green).

We were able to detect 17 (12%) captured genes that potentially originate from exon shuffling involving 36 parental genes (see Materials and methods). Among them, eight genes showed expression in at least one of the conditions analyzed. Then, we estimated the type of selection pressure applied to those genes that are likely functional by estimating the dN/dS ratio (*ω*) through sequence alignment with their closest homolog in *B. distachyon*. It revealed that most of the captured genes are under purifying selection, with a dN/dS ratio ranging from 0.2 to 0.6 (Figure 6). In contrast to the (*ω*) distribution observed for syntenic and nonsyntenic genes, we observed significantly larger dispersion for the captured genes (Fisher test, *P*-values <10^-5^), which indicates a more relaxed selection pressure on captured genes. In total, 19 captured genes (13%) were found to be expressed with a dN/dS ratio lower than 0.4.

Based on our findings on the 3B chromosome, we estimated that approximately 2,500 CACTA-captured gene/pseudogenes would be found at the whole genome scale, a range similar to the 1,194 genes captured by Helitron in maize [21] and 2,809 by Pack-MULE in rice [23].

## Discussion

### A fine-tuned strategy for constructing accurate models of transposable elements in a highly complex genome

Annotating TEs automatically and precisely in complex genomes is a challenge. However, high-quality TE annotations are necessary, not only for performing evolutionary analyses and better understanding the impact of TEs on genome organization and expression, but also to prevent mis-prediction of genes with cellular functions [29]. Here, we developed a strategy to overcome some of the problems due to the over-fragmentation of the predictions usually observed during TE modeling in large genomes. We used the knowledge accumulated during the past decades [31,34,36,49] to fine-tune our algorithm and automatically reconstruct nested clusters that can still be identified. With CLARI-TE, we were able to predict a set of 56,488 intact TEs on chromosome 3B. Half of the large intact TEs (>5 kb) were initially predicted as several truncated fragments through similarity search, revealing that for the time being curation is absolutely required to precisely delineate TEs in complex genomes. Moreover, among the 21,165 insertions of LTR-RTs that were used to study the dynamics of insertion/deletion along chromosome 3B, 30% were nested and, thus, would have been missed without automated joining by CLARI-TE. The use of our program prevented biasing the data towards recent TE insertions as usually observed in signature-based approaches [10,44]. Finally, with a curated TE library, this approach can be applied to any complex genomes (>50% TEs) with nested TEs.

The proportion of complete elements was lower than estimated previously in our pilot study on selected BACs from chromosome 3B [31]: they represent 22% and 6% for LTR-RTs and CACTAs, respectively, compared with 59% and 47% in the previous manually curated annotation. Similar observations have already been reported in maize, where the ratio of truncated to intact elements was estimated to be 0.5:1 [50], while it was 27:1 in the annotation of the reference genome sequence [47]. In both cases, the proportion of gaps, which is a reflection of the quality of the assembly, is the main limiting factor. Indeed, there are still 40,459 gaps in the chromosome 3B pseudomolecule, of which 57% were included in a TE by CLARI-TE, while the others prevented recovery of the surrounding TE structure. About 85% of the TEs surrounding a gap were annotated as truncated. In addition, CACTAs are the largest wheat TEs (up to 30 kb), are highly variable, and contain tandem repeated motifs, features that make them the most challenging superfamily to identify automatically.

Estimations of TE composition or organization of complex genomes are highly dependent on the reference TE library used as a basis for similarity search. In this study, similarity search-based annotation led us to assign 85% of the 3B sequence to TEs. Then, a *de novo* repeat identification allowed us to classify an additional 3% of the sequence to newly discovered repeats. Such a low value revealed that the ClariTeRep library is an almost exhaustive representation of the TE diversity present on the 3B chromosome, suggesting that downstream analyses were not biased by a lack of knowledge regarding the TE composition of the chromosome. Although the three homoeologous wheat subgenomes are similar in size and, thus, have similar proportions of TEs, this proportion appeared substantially higher on chromosome 3B than the 66% and 67% estimates obtained for the diploid genomes of *T. urartu* (AA) [39] and *Ae. tauschii* (DD) [40]. Such a difference highlights the strong impact of the methodology used to annotate TEs, rather than biological significance. This is supported by the results obtained after applying our TE modeling approach to these two draft genome sequences and the finding that TEs represent 77% and 74% of them, respectively, that is, proportions that are closer to what we have found for chromosome 3B. The main difference is related to the lack of knowledge regarding CACTAs in the reference TE library. Here, we predicted two to three times more CACTAs than previously suggested (5.44% for *T. urartu*, and 6.01% for *Ae. tauschii*), confirming the impact of the reference TE library on the biological interpretations. Finally, differences in TE proportions found between the diploid genomes and chromosome 3B are probably due to lack of sequence in the A and D draft genome sequences. With estimated sizes of 5.5 Gb and 5 Gb for *T. urartu* [39] and *Ae. tauschii* [40,51], respectively, TEs should represent 80 to 82% of the genome, considering that low copy DNA represents 1 Gb of the genome [42].

### Transposable element organization and dynamics

The 774 Mb 3B pseudomolecule represents the largest chromosome sequence ever assembled into one pseudomolecule. The uneven distribution of recombination rate, gene density, gene expression pattern, and TEs along the chromosome highlighted a striking partitioning with five distinct regions: a centromeric/pericentromeric region with the highest TE density and in which recombination is suppressed; two subtelomeric regions with the lowest TE density and where recombination mainly occurs; and two internal parts of chromosomal arms with intermediate features [42]. Behind the static view, the detailed study of TEs provided a dynamic view and novel insights into the evolutionary forces that have shaped this partitioning. First, we observed that LTR-RTs are the main contributors to the uneven distribution of TEs along the chromosome, a common feature of complex genomes like that of maize [47]. In contrast to other grass genomes where TEs have mainly transposed recently, we found a major amplification period 1.5 MYA that has been followed by a period of silencing and/or of increased loss from 1.0 MYA until now, confirming that the two hybridization events that led to hexaploid wheat did not trigger massive activation of transposition. Previous studies in wheat have shown a TE transposition burst immediately after allopolyploidization [36,52], but as suggest by Parisod *et al*. [53], this is probably a phenomenon restricted to specific TE families, and to mostly young, active TE populations. The availability of 21,165 complete LTR-RTs allowed us to study the TE activity at a scale not reached so far, revealing that each family had its own period of activity. Thus, overall, the B genome has been shaped by a succession of transposition waves from different families, some of which have escaped silencing, rather than by massive reactivation of all TEs simultaneously. Similar waves of amplification have already been described, such as in soybean [44], and are obviously observed in genomes where LTR-RTs have not been quickly eliminated.

The distribution of the TE insertion time along the chromosome showed an uneven pattern that is mainly due to the differential location of old LTR-RTs (those >3 MYA). In contrast, recently inserted elements (<1 MYA) exhibited a much more even distribution, suggesting that transposition occurred at similar rates along the chromosome and that the decrease of TE density towards the telomeres rather reflects a rapid elimination in the recombinogenic distal regions. Similar conclusions were suggested for the sorghum [12] and maize [47] genomes, while for more compact genomes, such as that of *Arabidopsis*, the enrichment of LTR-RTs in the centromeric regions has been explained by selection against the insertion of disruptive TEs in gene-rich regions [54]. Additional evidence for the more rapid elimination of TEs at the chromosome ends includes the overrepresentation of solo-LTRs and truncated LTR-RTs. This suggests that unequal homologous recombination (generating solo-LTRs) and illegitimate recombination (generating truncated TEs) are more frequent in the distal recombinogenic regions, as observed in rice [18]. In contrast to LTR-RTs, the density of class II DNA transposons increased in the distal regions and is positively correlated with both gene density and recombination rate. Such correlation was also observed in sorghum [12] and maize [4] but not in rice [18]. Non-autonomous DNA transposons are well known to be associated with genes [55,56] and their role in the regulation of gene expression has been suggested [8,57]. In wheat, with the exception of CACTAs, DNA transposons are shorter than class I elements and their insertion into gene-rich regions might be counter-selected at a lower frequency. In addition, the faster TE turnover in the distal regions suggested above could also explain the increase in DNA transposon density in these regions as a simple consequence of the compaction of the intergenic space that is mostly shaped by LTR-RTs. This differential deletion rate may also explain the increased TE diversity. Such a pattern was described in maize where the centromere was perceived as an environment settled only by ‘individuals’ that were most adapted to proliferate, creating a diversity-poor ecosystem [47]. Finally, preferential TE insertion seems to be a potential driving force towards the partitioning of chromosome 3B, with seven *gypsy* families having a chromodomain-containing integrase [13] significantly concentrated in the proximal region.

### Impact of CACTA transposons on gene duplication in wheat

DNA transposons represent 18% of the 3B chromosome sequence, which is the highest proportion observed among the sequenced grass genomes so far. CACTAs are the main contributors, representing 16% while they account for only 2.2% to 5.9% in sorghum, rice, *Brachypodium*, maize and barley [4,11,46,58]. This supports the hypothesis that CACTAs have been amplified specifically in the wheat lineage. Generally, genome size is mainly correlated with class I elements - for example, LINEs in human [59] and LTR-RTs in plants [60,61] - because of their copy-and-paste transposition mechanism allowing an increase in number in a short time period [62,63]. Thus, among plants, the wheat genome appears as a rare example of a massive amplification of cut-and-paste transposons.

Interestingly, 22 CACTA families were found to be preferentially associated with nonsyntenic genes, that is, genes that have been relocated via recent duplication events to a new chromosomal location. Previous studies in soybean [64], sorghum [12], and *Ipomoea tricolor* [65] have shown that CACTAs can capture genes and gene fragments. Here, we identified 145 CACTA-captured genes on chromosome 3B (2% of the gene content). Although most of them were gene fragments, as already observed at a smaller scale [66], 13% were both transcribed and under purifying selection, suggesting they are functional, and 12% likely originating from exon shuffling. Beyond gene capture mechanisms, CACTAs were proven to mediate gene duplication in wheat through double strand break repair created at the time of insertion [66]. Preferential association of CACTAs with nonsyntenic genes might reflect a higher rate of gene duplication, due to the high frequency of CACTA insertion. This study highlights the importance of CACTAs for gene duplication and the creation of new genes that may be associated with the adaptation of wheat to various environments [42]. Similar examples involving class II transposons have been described with Mutators in rice [23,67] and Helitrons in maize [21,22]. Therefore, it seems that superfamilies involved in gene capture have a tendency to proliferate and to be evolutionarily successful.

## Conclusion

In this study, we annotated TEs and their nested pattern in one of the most complex genomes. Our automated procedure significantly improved the accuracy of TE predictions compared with a classical similarity-search approach. Such a high quality annotation enabled us to determine and analyze the pattern of TE insertion, diversity and insertion time and revealed that the partitioning of the chromosome is mainly governed by higher deletion rates that are faster in recombining regions. We unraveled an unexpected abundance of CACTAs, and found a significant association with recently duplicated genes, suggesting a major impact of these elements on genome plasticity via the creation of genes. Such a mechanism may have provided wheat with an advantageous capability of adapting to a wide range of environments.

## Materials and methods

### Establishment of a classified library of *Triticeae* transposable element sequences

A library of TE sequences dedicated to similarity search-based annotation of TEs in the wheat genome was built as described in Choulet *et al*. [42]. Briefly, we retrieved 3,159 known full-length TE sequences, that is, elements having terminal repeats (terminal inverted repeats or LTRs) and/or features typical of complete LINEs*/*SINEs. We built 16 groups corresponding to each superfamily, and small non-autonomous MITEs were grouped separately from their autonomous counterparts to avoid computing multiple alignments with sequences of very different sizes. For each of the resulting 16 groups, an all-by-all BLAST [68] comparison (without filtering out low complexity sequences) was performed. BLAST output was analyzed with MCL (option -I 1.2) [69] in order to build clusters of sequences sharing similarity. The -I option, controlling the cluster granularity, was set to 1.2 for ‘very coarse grained clustering’, meaning that large clusters were built at that stage. These clusters were used to define the family level. Families of three or more members were considered for computing a multiple alignment using MAFFT (default parameter) [70]. A manual curation step was then applied. We used Jalview [71] as a visualization tool for the manual curation of the multiple alignments and their corresponding neighbor joining tree. Sequences introducing mistakes in the multiple alignments (due to inversions, deletions, or insertions) were identified and discarded so that all alignments were corrected. In addition, since MCL grouped sequences within large clusters, we identified the clearly separated monophyletic groups (according to the neighbor joining tree) among each individual family and, therefore, defined variants within the family; for instance, family RLG_famc8 is composed of three variants called RLG_famc8.1, RLG_famc8.2, and RLG_famc8.3. We named the library ClariTeRep and it is available upon request.

### Estimating the accuracy of transposable element prediction

We used a scaffold of 904 kb from the wheat chromosome 3B that does not correspond to a previously known region of the genome as a test sequence to estimate the accuracy of the TE modeling. This reference scaffold carries 196 TEs covering 91% of the sequence, with 47 nested insertions. To automate the comparison of the automated versus manually curated TE predictions and the calculation of sensitivity and specificity values, we developed compareAnnotTE.pl*.* Sensitivity and specificity were both estimated at three different levels: nucleotide, feature, and nested feature. At the nucleotide level, each nucleotide was considered to calculate sensitivity and specificity. At the feature level, only TE borders (all segments of TEs split into several pieces by nested insertions) were considered. At the nested feature level, the program considers borders of nested TEs to estimate the accuracy of the reconstruction of nested clusters. At the feature and nested feature levels, a predicted feature was considered as true positive if its borders correspond to the manually curated TE positions in a range of 10 bp.

### Similarity search and automated curation using CLARI-TE

We applied our procedure to the 2,808 scaffolds assembled for the 3B chromosome (HG670306 and CBUC010000001-CBUC010001450), and the *T. urartu* [39] and *Ae. tauschii* genomes [40]. Each sequence was investigated for TE content using RepeatMasker (cross_match engine with default parameters) [72] with ClariTeRep. We developed CLARI-TE, a perl program [73,74], to correct the raw similarity search results. It performs the three following steps. First, resolution of overlapping predictions. To solve the overlap between two predictions, priority was given to keep the prediction that covers an extremity of a TE. If none or both of the predictions cover a TE extremity, priority was given to keep the longest prediction and recalculate the positions of the other one. Second, merging predictions. Fragmentation of the TE models is due to the presence of gaps in the scaffolds and to the fact that a newly identified TE copy may diverge from the reference element so that one element is not predicted as a single piece but is rather split into several pieces matching different parts of elements from the same family. In that case, all neighbor pieces related to the same family were merged into a single feature if the collinearity of the matching segments was respected, except for LTR matching segments. LTR positions of reference TEs were annotated in our library and this information was considered during the merging process. Third, reconstruction of nested TEs. We developed a procedure to join separated features that are part of the same TE and have been split by nested insertions. Joining was allowed when two segments matching the same family (with respect of the collinearity between the prediction and the reference TE) are separated by a maximum of 10 predicted TEs. The final stage of the annotation is the assignment of intact full-length versus fragmented TEs. Intact full-length TEs are predictions covering at least 90% of the reference complete TE in the library and for which both extremities were identified (in a range of 50 nucleotides). Moreover, PFAM domains were searched for every complete element to detect chromodomains and transposase-like domains.

### Estimation of LTR-RT insertion date and phylogeny

We used the program TRsearch from REPET [45] to find the positions of both 5′ and 3′ LTRs from a complete element. We discarded predicted LTRs that did not correspond to the extremity of an element (in a range of 50 bp). Pairs of LTRs were aligned using MUSCLE [75] and insertion dates of LTR-RTs were estimated considering a mutation rate of 1.3 × 10^-8^ substitutions/site/year [76]. Finally, using R, the distribution of insertion dates was plotted for each family with 20 or more copies with an estimated date. For each distribution, a burst peak date was determined and a period of activity was calculated by considering the shortest period of time containing more than 80% of the dated insertions. Phylogeny was established by computing multiple alignments of all intact LTR-RTs belonging to families RLG_famc1 and RLC_famc2 using MAFFT [70]. A neighbor-joining tree was then built using FasTree [77]. The tree was drawn using figtree 1.4.2 [78].

### Distribution of transposable elements along the chromosome

The distribution of TEs along the chromosome 3B sequence was computed by calculating the proportion (in size) and number of TEs in a sliding window of 10 Mb with a step of 1 Mb. The distribution of the TE diversity along the chromosome was computed using the same window by calculating the number of families per window. To prevent drastic variations due to mis-predictions, we considered the number of families representing 99% of the TE fraction per window (N99).

### Prediction of solo-LTRs

Based on of the 30,406 intact LTR-RTs predicted on chromosome 3B, we built a library of LTR sequences by extracting 18,928 LTRs flanked by canonical 5′-TG and 3′-CA dinucleotides. This library was used for an additional round of similarity searching using RepeatMasker on the full chromosome 3B. To distinguish solo LTRs from truncated LTR-RTs, we searched specifically for the presence of a 5 bp target site duplication (one nucleotide variation tolerated) flanking the matching region.

### Hierarchical clustering of distributions

In order to identify CACTA families with similar distributions along the chromosome, we performed a hierarchical clustering of the distributions calculated in a sliding window of 10 Mb (step of 1 Mb) by using the R package ‘pvclust’ [79]. We considered only families representing at least 0.01% in at least one 10 Mb window. The Pearson correlations were calculated between each pair of distribution and a clustering was applied by the agglomerative method ‘average’ (N = 10,000 bootstrap resampling).

### Transposable element abundance in the vicinity of genes

The CDS positions of the annotated chromosome 3B protein-coding genes were used to estimate the relative abundance of TE families in the 20 kb upstream and downstream regions. The average proportion of a given TE family was calculated for each nucleotide position in the CDS surrounding sequence by considering the orientation of the genes.

### Detection of transposable element-captured gene and exon shuffling events

In order to detect TE-captured genes, we isolated genes flanked by two elements belonging to the same family, as a trace of a potential capture event. A total of 558 potential gene capture events were detected, 235 (42%) involving a CACTA, 104 (19%) involving a *gypsy*, and 219 (39%) involving other superfamilies. We manually checked the presence of target site duplications as evidence of gene capture.

To decipher potential exon shuffling events, we searched for the presence of chimeric genes. A similarity search using BLASTP against the *B. distachyon* proteome was performed for each captured gene product. Wheat proteins aligning over 70% of their length to a *B. distachyon* protein were filtered out. For the others, we develop a parsing procedure to solve the overlapping regions of similarity between a *B. distachyon* protein and our query sequence and to detect chimeric proteins, that is, proteins showing non-overlapping segments of similarity with different *B. distachyon* proteins.

### Data availability

Sequences and annotations of the reference pseudomolecule and unassigned scaffolds have been deposited in the European Nucleotide Archive (ENA; project PRJEB4376) under accession numbers HG670306 and CBUC010000001 to CBUC010001450, respectively. The source code of the CLARI-TE program is available at [74].

## Additional file

Additional file 1:
**The following additional data are available with the online version of this paper. Table S1.** TE composition (in percentage of the sequence) of bread wheat chromosome 3B and of the draft genome sequences of *Triticum urartu* and *Aegilops tauschii*. **Table S2.** Correlation between TE content, genetic recombination rate and gene density. **Figure S1.** Distribution of the average percentage of similarity shared among LTR-RT families for *Brachypodium* (bd), rice (os) sorghum (sb), maize (zm) and wheat (ta). **Figure S2.** Dendrograms of two families of LTR-RTs: RLG_famc1 **(A,B)** and RLC_famc2 **(C,D)**. **Figure S3.** Distribution of the number of different TE families found in a 10 Mb window sliding along the wheat chromosome 3B (in Mb). **Figure S4.** Proportions of solo-LTRs, truncated TEs, and recombination rates in three different regions of chromosome 3B. **Figure S5.** Preferential location of gypsy families along the 3B chromosome.

## Abbreviations

BAC: bacterial artificial chromosome
CDS: coding sequence
LINE: long interspersed nuclear element
LTR: long terminal repeat
LTR-RT: long terminal repeat retrotransposon
MITE: miniature inverted-repeat transposable element
MYA: million years ago
SINE: short interspersed nuclear element
TE: transposable element
TREP: *Triticeae* REPeat sequence database
