# Supplementary material for: Organization and evolution of transposable elements along the bread wheat chromosome 3B
Source: Genome Biol. 2014 Dec 3;15(12):546. doi: 10.1186/s13059-014-0546-4 (PMC4290129; doi:10.1186/s13059-014-0546-4)
Supplement: Additional file 1: — The following additional data are available with the online version of this paper. Table S1. TE composition (in percentage of the sequence) of bread wheat chromosome 3B and of the draft genome sequences of Triticum urartu and Aegilops tauschii. Table S2. Correlation between TE content, genetic recombination rate and gene density. Figure S1. Distribution of the average percentage of similarity shared among LTR-RT families for Brachypodium (bd), rice (os) sorghum (sb), maize (zm) and wheat (ta). Figure S2. Dendrograms of two families of LTR-RTs: RLG_famc1 (A,B) and RLC_famc2 (C,D). Figure S3. Distribution of the number of different TE families found in a 10 Mb window sliding along the wheat chromosome 3B (in Mb). Figure S4. Proportions of solo-LTRs, truncated TEs, and recombination rates in three different regions of chromosome 3B. Figure S5. Preferential location of gypsy families along the 3B chromosome. [file 13059_2014_546_MOESM1_ESM.pptx]

## Slide 1
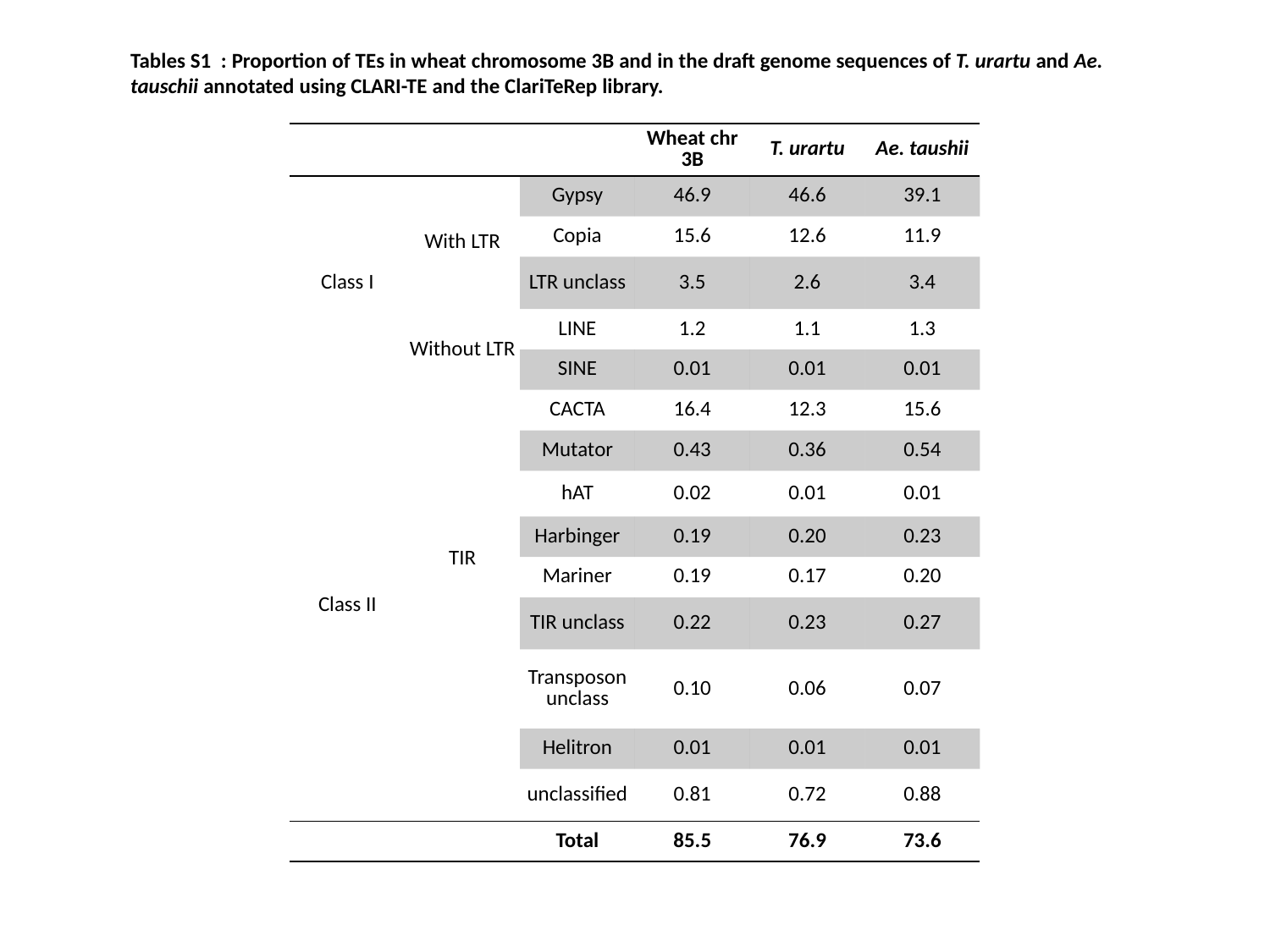

Tables S1 : Proportion of TEs in wheat chromosome 3B and in the draft genome sequences of T. urartu and Ae. tauschii annotated using CLARI-TE and the ClariTeRep library.
| | | | Wheat chr 3B | T. urartu | Ae. taushii |
| --- | --- | --- | --- | --- | --- |
| Class I | With LTR | Gypsy | 46.9 | 46.6 | 39.1 |
| | | Copia | 15.6 | 12.6 | 11.9 |
| | | LTR unclass | 3.5 | 2.6 | 3.4 |
| | Without LTR | LINE | 1.2 | 1.1 | 1.3 |
| | | SINE | 0.01 | 0.01 | 0.01 |
| Class II | TIR | CACTA | 16.4 | 12.3 | 15.6 |
| | | Mutator | 0.43 | 0.36 | 0.54 |
| | | hAT | 0.02 | 0.01 | 0.01 |
| | | Harbinger | 0.19 | 0.20 | 0.23 |
| | | Mariner | 0.19 | 0.17 | 0.20 |
| | | TIR unclass | 0.22 | 0.23 | 0.27 |
| | | Transposon unclass | 0.10 | 0.06 | 0.07 |
| | | Helitron | 0.01 | 0.01 | 0.01 |
| | | unclassified | 0.81 | 0.72 | 0.88 |
| | | Total | 85.5 | 76.9 | 73.6 |

## Slide 2
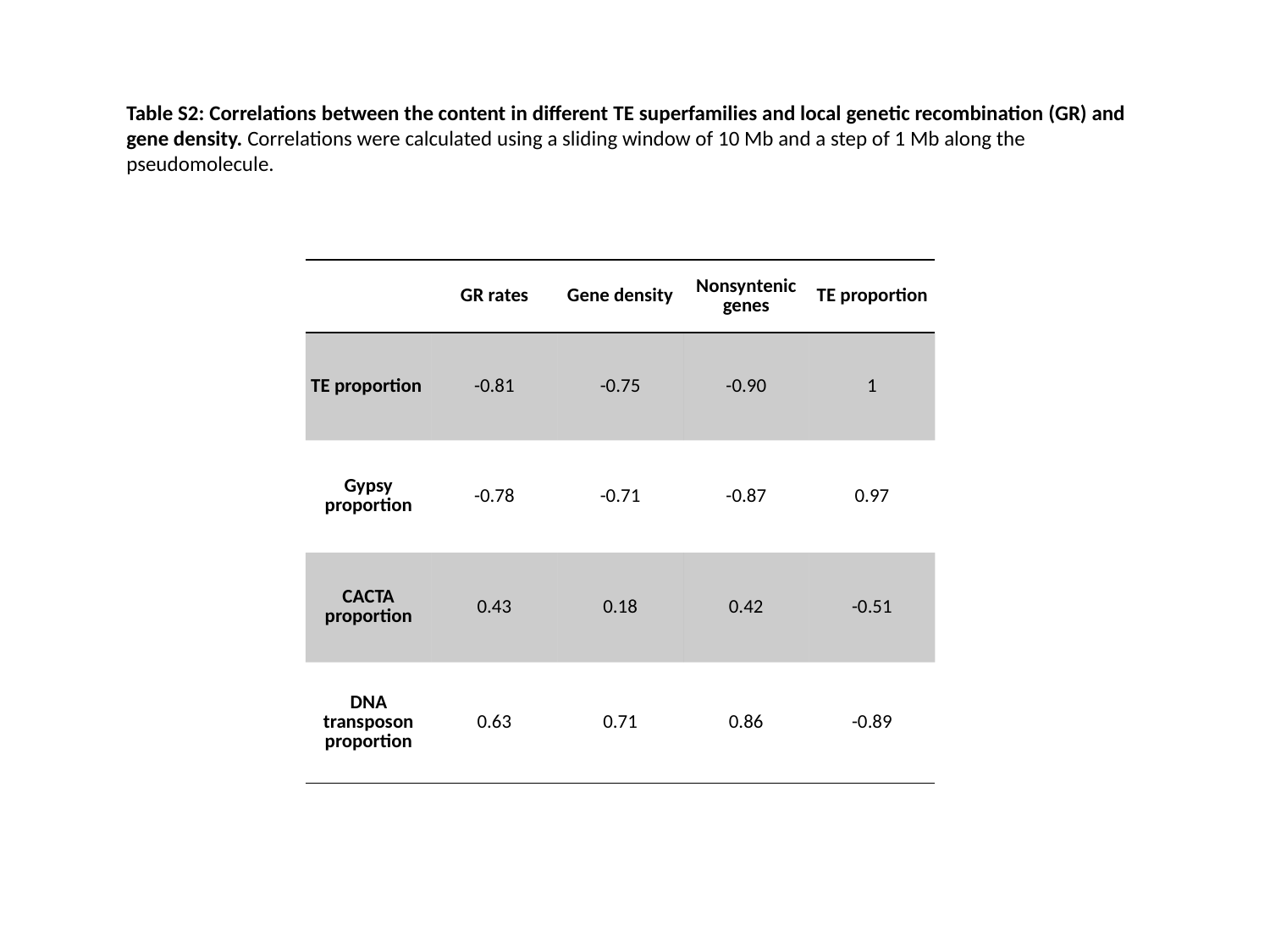

Table S2: Correlations between the content in different TE superfamilies and local genetic recombination (GR) and gene density. Correlations were calculated using a sliding window of 10 Mb and a step of 1 Mb along the pseudomolecule.
| | GR rates | Gene density | Nonsyntenic genes | TE proportion |
| --- | --- | --- | --- | --- |
| TE proportion | -0.81 | -0.75 | -0.90 | 1 |
| Gypsy proportion | -0.78 | -0.71 | -0.87 | 0.97 |
| CACTA proportion | 0.43 | 0.18 | 0.42 | -0.51 |
| DNA transposon proportion | 0.63 | 0.71 | 0.86 | -0.89 |

## Slide 3
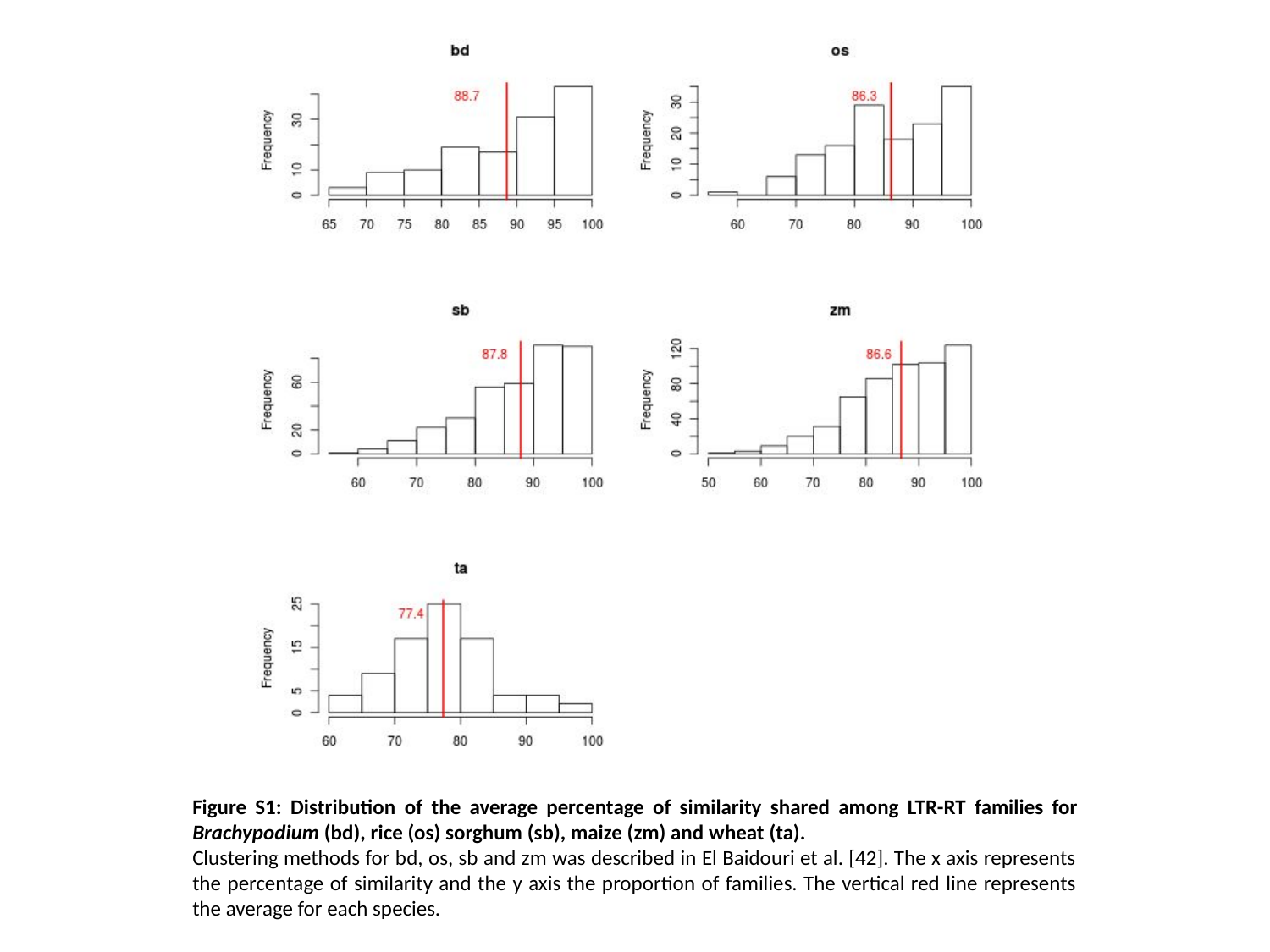

Figure S1: Distribution of the average percentage of similarity shared among LTR-RT families for Brachypodium (bd), rice (os) sorghum (sb), maize (zm) and wheat (ta).
Clustering methods for bd, os, sb and zm was described in El Baidouri et al. [42]. The x axis represents the percentage of similarity and the y axis the proportion of families. The vertical red line represents the average for each species.

## Slide 4
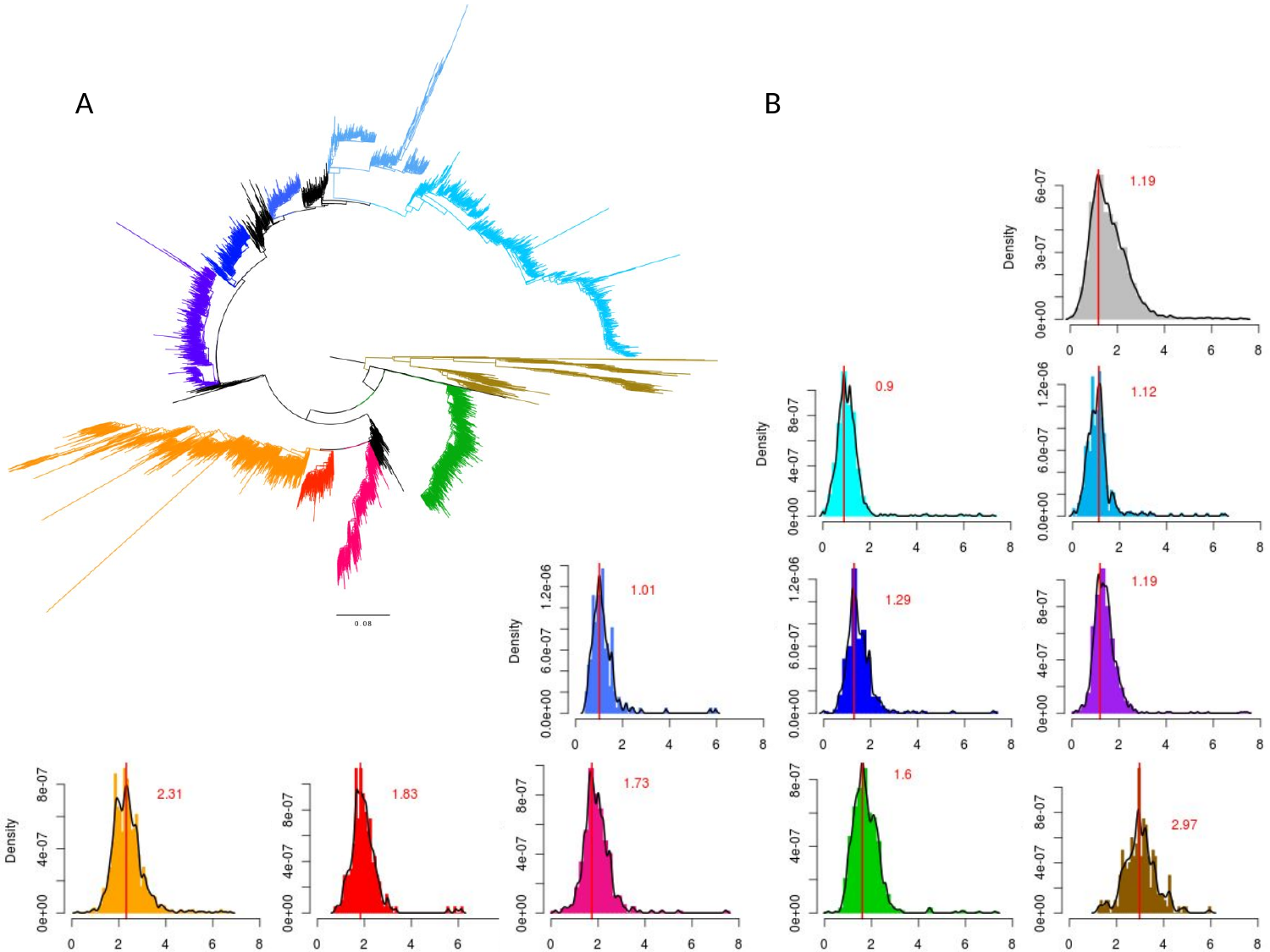

A
B

## Slide 5
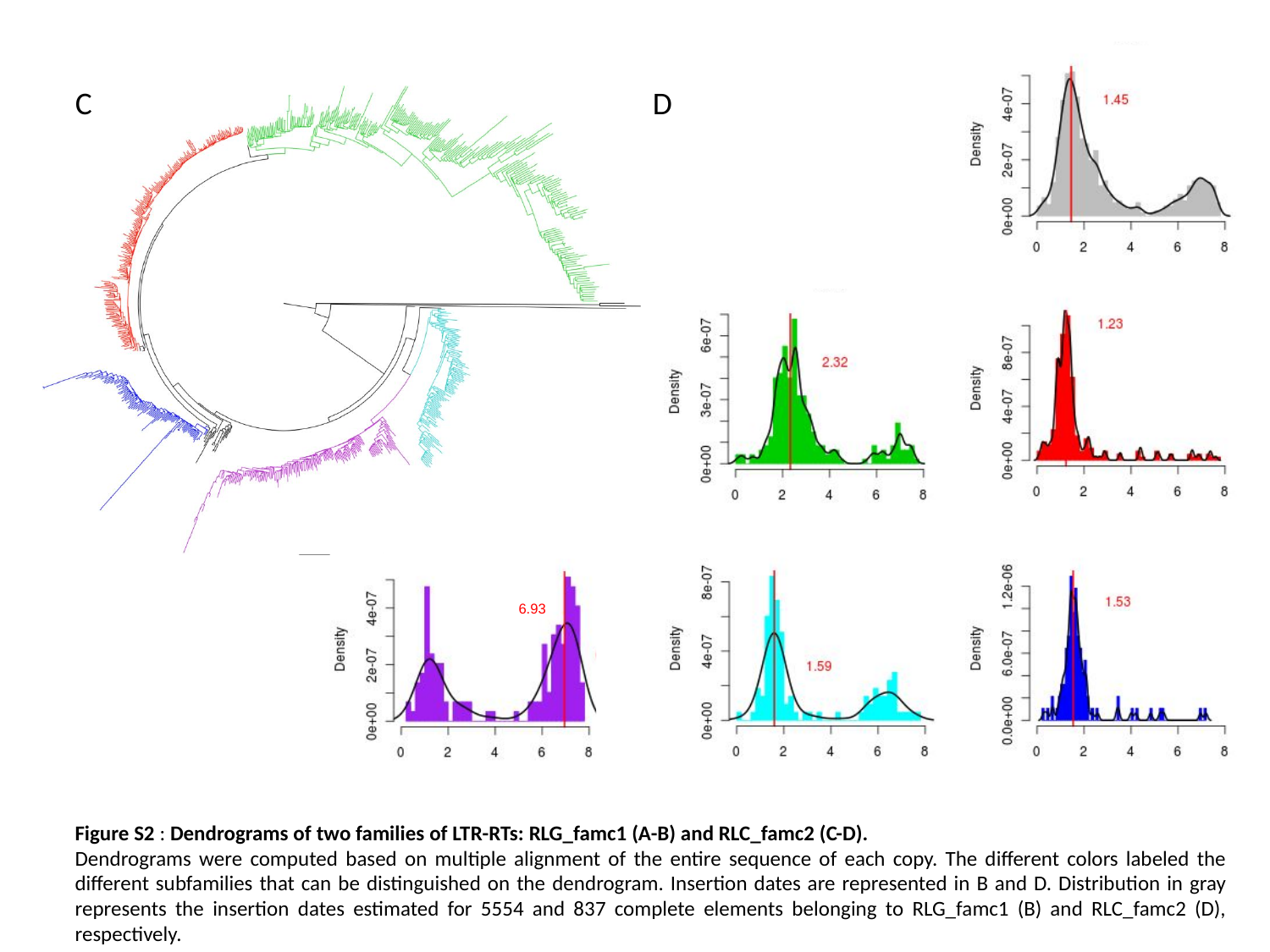

C
D
6.93
Figure S2 : Dendrograms of two families of LTR-RTs: RLG_famc1 (A-B) and RLC_famc2 (C-D).
Dendrograms were computed based on multiple alignment of the entire sequence of each copy. The different colors labeled the different subfamilies that can be distinguished on the dendrogram. Insertion dates are represented in B and D. Distribution in gray represents the insertion dates estimated for 5554 and 837 complete elements belonging to RLG_famc1 (B) and RLC_famc2 (D), respectively.

## Slide 6
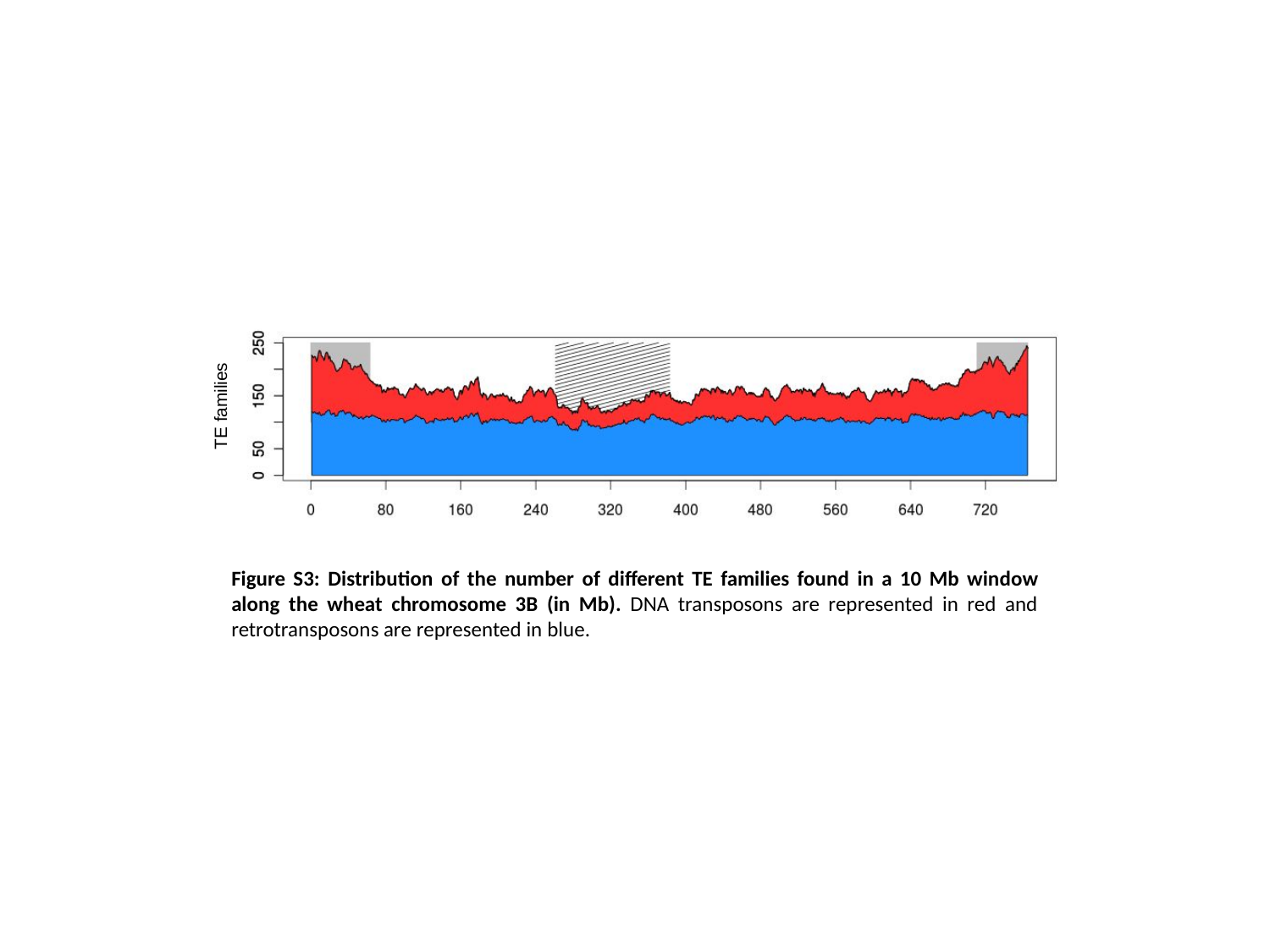

TE families
Figure S3: Distribution of the number of different TE families found in a 10 Mb window along the wheat chromosome 3B (in Mb). DNA transposons are represented in red and retrotransposons are represented in blue.

## Slide 7
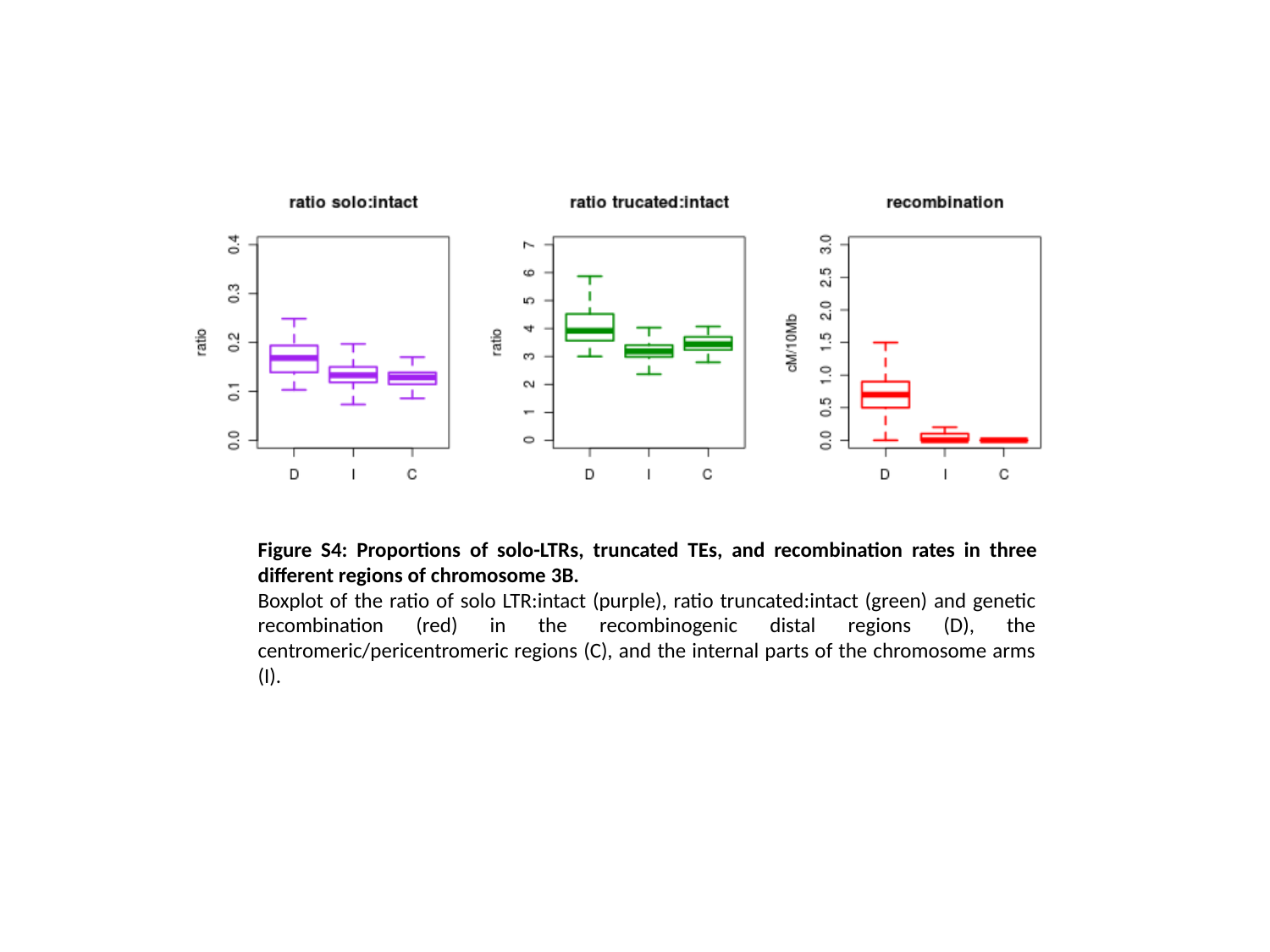

Figure S4: Proportions of solo-LTRs, truncated TEs, and recombination rates in three different regions of chromosome 3B.
Boxplot of the ratio of solo LTR:intact (purple), ratio truncated:intact (green) and genetic recombination (red) in the recombinogenic distal regions (D), the centromeric/pericentromeric regions (C), and the internal parts of the chromosome arms (I).

## Slide 8
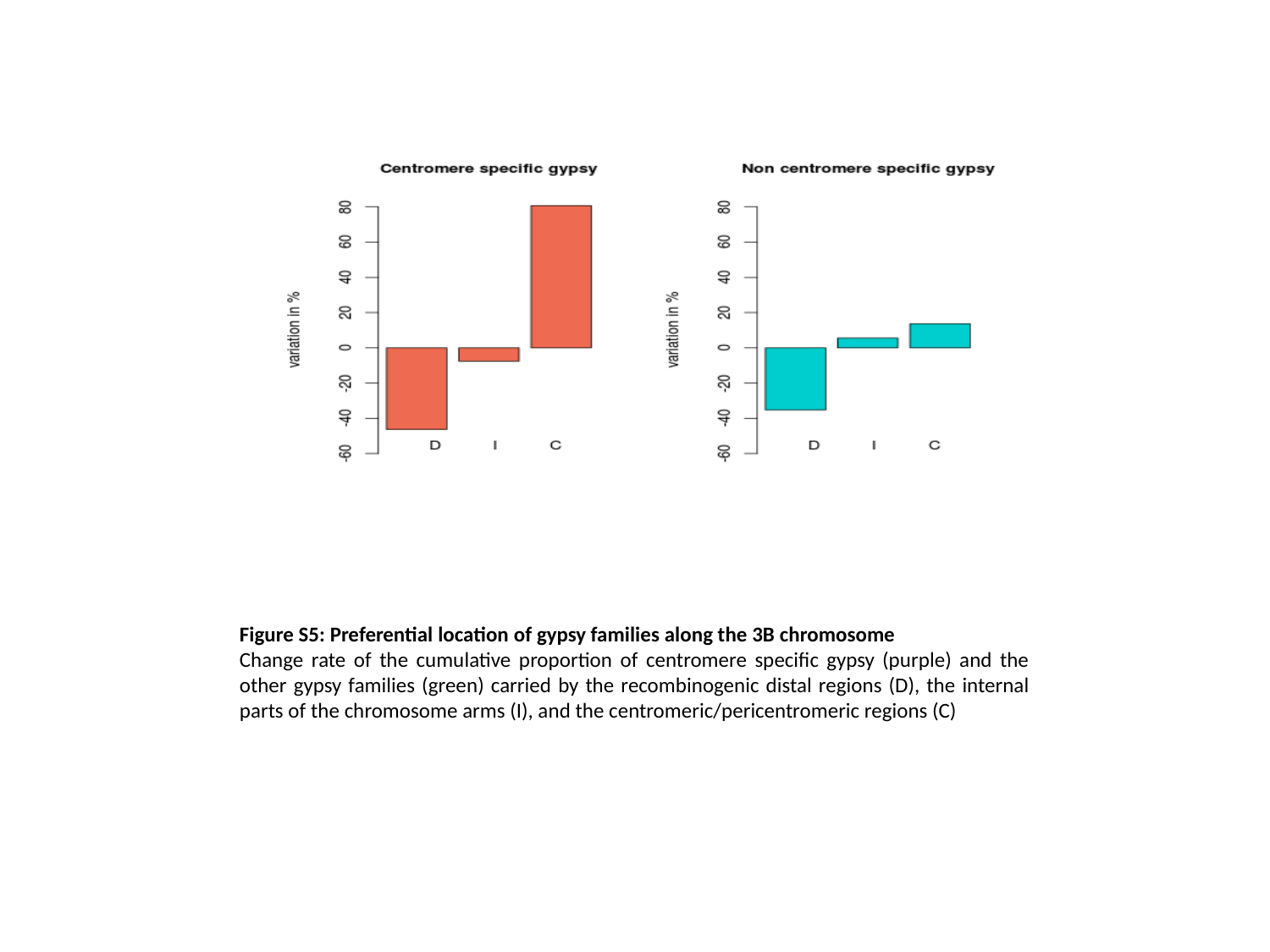

Figure S5: Preferential location of gypsy families along the 3B chromosome
Change rate of the cumulative proportion of centromere specific gypsy (purple) and the other gypsy families (green) carried by the recombinogenic distal regions (D), the internal parts of the chromosome arms (I), and the centromeric/pericentromeric regions (C)
